# Une étude cas-témoins pour déterminer les facteurs de non-observance du suivi médical chez les patients diabétiques à Kinshasa, en 2010

**DOI:** 10.11604/pamj.2014.17.258.2892

**Published:** 2014-04-08

**Authors:** Kennedy Mense, Mala Ali Mapatano, Paulin Beya Mutombo, Marie Claire Muyer

**Affiliations:** 1 Institut Supérieur des Techniques Médicales de Bosobe, Bosobe, République Démocratique du Congo; 2 Ecole de Santé Publique, Centre National d'Epidémiologie du Diabète, Kinshasa, République Démocratique du Congo

**Keywords:** Diabète sucré, non observance, suivi médical, coöts du suivi médical, revenu des ménages, diabetes mellitus, noncompliance, medical monitoring, costs of medical monitoring, household income

## Abstract

**Introduction:**

Le diabète est un problème majeur de santé publique et un fardeau économique mondial qui n’épargne pas la RD-Congo. Bien que sa prise en charge soit codifiée, la plupart des diabétiques n'arrivent pas à respecter les rendez-vous de suivi. Cette étude vise principalement à identifier les déterminants de la non-observance du suivi médical chez les diabétiques à Kinshasa.

**Méthodes:**

Il s'agit d'une étude cas-témoins où les cas sont les patients diabétiques non observant le suivi médical et les témoins, ceux répondant régulièrement au suivi médical. Couvrant la période du 1erjanvier au 31 décembre 2010, l’étude a porté sur un échantillon aléatoire de 154 sujets répartis entre 77 cas et 77 témoins.

**Résultats:**

Les données indiquent une association entre la non-observance du suivi médical et le revenu (niveau de vie) des ménages. Les cas provenant des ménages à faible revenu courent six fois plus le risque d’être non-observants. Par contre, entre le niveau de connaissance et la non-observance l'association notée n’était pas statistiquement significative. Le respect des rendez-vous pourrait être amélioré de 77% si le revenu des ménages des diabétiques était augmenté. Le coût total mensuel du suivi médical est estimé à 27,2 USD, alors que le revenu permanant des ménages se situe à 306,6 USD.

**Conclusion:**

Le bas niveau de vie mais pas celui de l'ignorance est un déterminant de la non-observance des visites de suivi du malade diabétique.

## Introduction

Le diabète constitue un problème majeur de santé publique dans le monde. Les estimations de l'OMS et de la Fédération Internationale de diabète (FID) montrent qu'il prend les allures d'une pandémie dont les chiffres passent de 30 millions de cas dans le monde en 1985 [1] à 366 millions en 2011 [2]. Ce chiffre dépassera 552 millions d'ici 2030, si aucune intervention robuste ne se met pas en place [2].

En Afrique subsaharienne, le nombre de diabétiques en 2011 a été estimé à 14,7 millions, estimation qui se traduit à 737 090 cas en République Démocratique du Congo (RD-Congo) pour la même année [2]. A Kinshasa, une étude menée en 2000 a estimé la prévalence du diabète à 7% chez les adultes [3].

Toutefois, ces chiffres nous paraissent sous-estimés, car beaucoup de diabétiques ignorent leur état et certains sont suivis dans des structures privées ou encore par des tradipraticiens dont les statistiques restent inconnues des services publics [4]. Il convient de préciser ici qu’à cause de ses multiples complications, le diabète est considéré comme une des principales causes de décès dans le monde. En effet, environ 4 millions de morts par an sont imputables au diabète [5]. Cependant, ces complications peuvent être prévenues ou réduites sensiblement par une bonne prise en charge et un suivi médical régulier [6].

Dans le souci d'améliorer la qualité de vie des malades, De Clerk [7], emboitant à cet égard le pas à la FID [8], recommande que tout diabétique soit suivi régulièrement à travers des visites mensuelles puis par une visite par an à l'occasion de laquelle un bilan complet doit être effectué. A cet effet, chaque patient dispose d'un carnet dans lequel les rendez-vous ainsi que d'autres informations en rapport avec la maladie sont repris. La fixation des rendez-vous tient compte de la disponibilité du patient. C'est dans ce cadre que le Bureau Diocésain des œuvres Médicales (BDOM) a implanté à travers la ville de Kinshasa, des cliniques diabétiques dans des hôpitaux et centres de santé [9].

Plusieurs études menées sur le suivi médical des malades diabétiques se focalisent sur la qualité du suivi [10]; ou bien sur le suivi thérapeutique [11–14]. Rares sont les auteurs qui s'intéressent à l'observance de ce suivi par les malades, aspect que cette étude vise d'explorer.

En fait, examiner le respect des rendez-vous par les malades présente un double intérêt. D'une part, rater les rendez-vous pourrait ajouter à la frustration du personnel de soins qui éprouve déjà des difficultés à gérer la surcharge des malades. D'autre part, le non-respect des rendez-vous influe négativement sur les soins du patient, lequel risque de manquer les opportunités de recevoir des médicaments ou de voir détecter à temps les complications de sa maladie. On constate, par exemple, que 67% des diabétiques pris en charge à l'Hôpital Général de Référence de Kisenso ne sont pas observants du suivi médical. Il en résulte que 53% de ces malades développent des complications cardio-vasculaires [15]. Cette situation est probablement identique dans l'ensemble du pays.

Cependant, nous avons restreint notre étude au réseau BDOM, desservant la partie Ouest de Kinshasa (Kin-Ouest), pour examiner les facteurs de la non-observance des patients diabétiques du suivi médical. Notre recherche a été guidée par deux hypothèses à savoir: la non-observance du suivi médical chez les diabétiques dans les formations médicales de BDOM Kin-Ouest est due au faible revenu des ménages et; à l'ignorance des patients diabétiques sur le diabète et les moyens de prévention de ses complications. Alors, nous avons retenu les objectifs spécifiques suivants: Evaluer la capacité des diabétiques à faire face au coût du suivi médical dans les structures de soins du réseau BDOM Kin-Ouest; Identifier les facteurs de non-observance du suivi médical, en particulier vérifier le lien entre l'ignorance des patients sur le diabète avec le suivi médical.

## Méthodes

### Le cadre d’étude

L’étude porte sur les patients diabétiques rapportés en 2010 dans la partie ouest du réseau BDOM Kinshasa qui couvre les zones de santé de Mont-Ngafula, Lemba et Kisenso. Le réseau BDOM a été choisi parce qu'il prend en charge le plus grand nombre de diabétiques enregistrés dans la ville de Kinshasa. Le BDOM, est le service médical chargé de coordonner toutes les interventions initiées dans le secteur de la santé par l'Archidiocèse de Kinshasa. Au total, il a enregistré 5.874 diabétiques en 2010, soit 70% des patients dans la ville de Kinshasa [9].

### Le Type d’étude

Il s'agit d'une étude cas-témoins où les cas sont des patients diabétiques non observant le suivi médical et les témoins, les patients répondant régulièrement au rendez-vous. La période d’étude s'est étendue du 1er janvier au 31 décembre 2010.

### La population d’étude

L’étude a ciblé tous les malades diabétiques suivis dans les huit formations sanitaires du réseau BDOM Kin-Ouest, à savoir l'Hôpital Général de Référence de Kisenso, le Centre Hospitalier du Mont Amba, les centres de santé Elimo Santu, Mawagali, Lisungi, Mater Dei, Kindele et Révélation (Tableau 1).

**Tableau 1:** Distribution des malades diabétiques selon les formations sanitaires, Kin-Ouest, 2010

Structures sanitaires	Nombres de cas	Zone de santé
Centre de santé lisungi	87	Mont Ngafula
Centre de santé mawagali	63	Mont Ngafula
Centre de santé kindele	55	Mont Ngafula
Centre de santé mater dei	48	Mont Ngafula
Centre de santé elimo-santu	123	Lemba
Centre hospitalier de mont amba	56	Lemba
Centre de santé révélation	27	Lemba
Hôpital général de référence de Kisenso	91	Kisenso
**Total**	**550**	

***Source:*** Registres des malades des formations médicales, BDOM, Kin-Ouest, 2010

### Définitions des termes

**Suivi médical**: Il se rapporte au rythme de contrôle du patient par le service de soins de santé. Dans la perspective où le patient doit être vu chaque mois, le suivi a été jugé bon lorsque le patient s'est présenté au contrôle au moins huit mois sur douze. Le patient est donc dit « **observant** ». Autrement, il est dit « **non observant** ». Dans cette étude, l'observance est mesurée en examinant le carnet de soins. Cette variable est la variable dépendante.

**Revenu du ménage**: C'est une variable quantitative exprimée en USD. Dans cette étude menée dans un contexte d'une économie informelle, les dépenses mensuelles moyennes engagées par le chef du ménage sont utilisées comme indicateur du revenu permanent du ménage [16].

**Le niveau de vie**: La variable revenu du ménage a été dichotomisée pour qualifier les ménages: Faible niveau de vie pour ménage vivant avec moins de 1,25 USD par personne par jour; Le niveau de vie acceptable pour ménage vivant avec un revenu permanent supérieur ou égal à 1,25 USD par personne par jour. Le seuil de 1,25 USD est publié par PNUD depuis décembre 2010 [17].

**La connaissance du diabète**: Elle apprécie le degré d'information que possède le patient sur les signes cliniques, les complications et les moyens de prévention des complications du diabète. Le niveau de connaissance a été jugé bon lorsque le patient connait au moins la moitié des symptômes et signes cliniques majeurs du diabète (polyurie, polydipsie, amaigrissement, polyphagie, asthénie physique, sécheresse des lèvres); la moitié des complications du diabète (coma, paralysie, ulcère du pied, douleur au niveau des membres inférieurs, troubles visuels, atteinte des reins, du système cardiovasculaire) et la moitié des moyens de prévention de ces complications (consultations régulières, respect des mesures hygiéno-diététiques, régularité de traitement). En dessous de la moitié, la connaissance est jugée faible (ignorance).

**Niveau d'instruction**: représente le plus haut niveau d’études atteint par le patient. Dans le cadre de notre étude, en dessous du diplôme d’état (baccalauréat), le niveau d'instruction a été jugé faible. Il était satisfaisant si l'enquêté était détenteur au moins d'un diplôme d'Etat.

### La taille de l’échantillon

Notre échantillon était composé de 77 cas et 77 témoins (1 témoin pour 1 cas). La taille de l’échantillon était calculée à l'aide de la formule suivante: n ≥2(zα + z1-β)2xp (1-p) / (po-p1)2 où n: le nombre de cas à déterminer; α: seuil de signification;β:30%;zα = 1,282;z1-β = 0,53;Po:Proportions des témoins exposés;P1 Proportion des cas exposés;P:proportion sujets exposés dans les deux groupes (cas et témoins); OR: Odds ratio attendu = 2 Le test étant unilatéral,zα = z1-α Ainsi, n ≥ 2x (1,28 + 0,53)2x0,267x(1-0,267) / (0,20-0,333)2 = 71,344 = 72 Dans le but de palier le risque de non-réponses, l’échantillon a été ramené à 77 pour les cas et 77 pour les témoins.

### L’échantillonnage

A partir des dossiers des malades, nous avons répertorié tous les diabétiques non observants et avons calculé leur proportion dans chaque formation médicale. Puis nous avons multiplié cette proportion par la taille de l’échantillon pour obtenir la taille des sous échantillons pour chaque formation médicale. Nous avons ensuite procédé à un tirage aléatoire simple des cas que nous avons appariés aux témoins de même sexe, soignés dans la même structure sanitaire.

### Critère d'inclusion

Les cas étaient des diabétiques non-observants suivis dans les formations sanitaires du réseau BDOM Kin-Ouest, entre le 1erjanvier et le 31 décembre 2010. Les témoins étaient des diabétiques observants, suivis dans les mêmes formations sanitaires que les cas.

### La collecte des données

Les données ont été extraites des dossiers des malades. A l'aide d'un questionnaire, nous avons interviewé les malades ou leurs mères, pour les enfants, pour des informations supplémentaires. Deux contrôles de qualité étaient effectués au cours de la collecte pour vérifier la complétude, la précision et la fiabilité des réponses, quand l'enquêteur remettait le questionnaire et, au hasard, une fiche sur dix était ré-contrôlée par rapport au dossier et carnet du malade.

### Analyse des données

Après leur saisie et traitement en Epi Info, les données étaient analysées avec SPSS, version 16. Les tests de Khi-carré et de régression logistique (uni et multi-variée) ont été effectués pour vérifier la relation entre les facteurs de risque et la non-observance du suivi médical.

### Considérations éthiques

Ce travail a été réalisé dans le cadre d'un mémoire pour l'obtention d'une maitrise en Economie de la Santé. A cet effet, le Comité d’éthique de l’école de Santé Publique de Kinshasa a donnée l'autorisation.

## Résultats

Au total 77 cas et autant de témoins ont participé à l’étude. Le sexe féminin était prédominant dans les deux groupes. L’âge des patients diabétiques variait entre 21 et 82 ans (CI95%: 41-76 ans). La taille des ménages, dont la moyenne était de 7,2, a varié entre 2 et 17 personnes. Les cas étaient statistiquement plus susceptibles de provenir des ménages de grande taille (Tableau 2).

**Tableau 2:** Comparaison des variables d'intérêt entre les cas et les témoins

Variables		Cas (%)	Témoins (%)	OR*	95% CI		*P*
**Taille ménage**	≤ 3	2 (2,6)	10 (13,0)	1,00			
	4-6	31(40,3)	29 (37,7)	5,34	1,08	26,48	0,040
	7-9	27 (35,1)	24 (31,2)	5,63	1,12	28,27	0,036
	10-12	11 (14,3)	10 (13,0)	5,50	0,96	31,43	0,055
	≥13	6 (7,8%)	4 (5,2)	7,50	1,04	54,12	0,046
**Sexe**							
	Féminin	47 (61,0)	52 (67,5)				
	Masculin	30 (39,0)	25 (32,5)				
**Age**							
	≤ 40 ans	5 (6,5)	1 (1,3)	1,00			
	41-50 ans	12 (15,6)	8 (10,4)	0,30	0,03	3,07	0,310
	51-60 ans	18 (23,4)	27 (35,1)	0,13	0,01	1,24	0,076
	61-70 ans	32 (41,6)	29 (37,7)	0,22	0,02	2,00	0,179
	≥ 71 ans	10 (13,0)	12 (15,6)	0,17	0,02	1,67	0,128
**Niveau d’étude**							
	Primaire	33 (42,9)	25 (32,5)	1,00			
	Secondaire	22 (28,6)	34 (44,2)	0,49	0,23	1,03	0,061
	Universitaire	9 (11,7)	4 (5,2)	1,70	0,47	6,18	0,417
**Connaissance**							
	Faible	51 (66,2)	43 (55,8)				
	Acceptable	26 (33,8)	34 (44,2)	0,64	0,34	1,24	0,187
**Revenu**							
	≤ 54$	21(27,3)	16 (20,8)	1,00			
	55-104$	27 (35,1)	18 (23,4)	1,14	0,47	2,76	0,767
	105-154$	11 (14,3)	10 (13,0)	0,76	0,24	2,47	0,650
	155-204$	7 (9,1)	9 (11,7)	0,38	0,08	1,76	0,217
	205-254$	3 (3,9)	10 (13,0)	0,46	0,09	2,20	0,329
	≥ 255$	8 (10,4)	14 (18,2)	0,44	0,15	1,29	0,133
**Niveau de vie**							
	≥1,25$	26 (33,8)	34 (44,2)	1,00			
	<1,25$	51 (66,2)	43 (55,8)	4,3	2,2	9,5	0,000

Le test de Khi carré et la régression logistique univariée ont été utilisés pour étudier la relation entre les différentes variables et l'observance du suivi médical.

* OR non ajusté

Les cas étaient nombreux à n'avoir pas dépassé le niveau primaire (42,9%). Par contre la majorité des témoins avaient atteint le niveau secondaire (44,2%). Néanmoins, la différence n'est pas statistiquement significative. Il n'y a pas non plus de différence d’âge entre les cas et les témoins par rapport à l'observance. Le niveau de connaissance sur le diabète n’était pas statistiquement différent entre les deux groupes (OR= 0.64; P= 0,187). Pourtant, les cas (66,2%) ont montré une plus faible connaissance sur le diabète que les témoins (55,8%).

La proportion de ménages avec un revenu inférieur à 105$ était plus élevée chez les cas (62,4%) que chez les témoins (44,2%). Toutefois cette différence n'est pas statistiquement significative. Plus de la moitié des ménages vivaient en dessous de 1,25 UDS par jour. Dans une analyse non ajustée, les cas couraient quatre fois plus de risque que les témoins à vivre avec moins de 1,25$ par jour (OR= 4,3; CI95%: 2,2-9,5). La fraction du risque de la non-observance du suivi diabétique attribuable au faible revenu du ménage était de 77.3%.

Les dépenses mensuelles moyennes étaient estimées à 306,6 USD par ménage. La classe modale pour les cas était de 220-269$ tandis que celle des témoins était ≥ 270-319$. Deux tiers des cas (65%) dépensaient au plus 269$ tandis que 50,7% des témoins dépensaient au moins 270$. Cependant aucune différence statistiquement significative n'a été observée. Le coût direct du suivi médical des diabétiques a varié entre 8160FC (9 USD) et 17750FC (19.5 USD). Le coût direct moyen était estimé à 13587,50FC (14,9$) dont le coût des médicaments vient en tête avec près de 30% de la moyenne pour les deux groupes (Tableau 3). Le patient sous insuline dépensait presque deux fois plus (17 800 FC) que celui sous antidiabétiques oraux (9 375 FC), lors d'une visite de suivi. Le traitement par insuline coûtait plus de 4 fois plus cher (6 750 FC) que par antidiabétiques oraux (1 515 FC).

**Tableau 3:** Coûts directs à la dernière visite médicale

Rubriques	Coût moyen avec insulino-thérapie (FC)	Coût moyen avec Antidiabétiques oraux (FC)	Moyenne en FC
Consultation	1550 (8,7%)	1750 (18,7%)	1625 (12%)
Laboratoire	3100 (17,4%)	2600 (27,7%)	2850 (21,1%)
Autres examens	4850 (27,3%)	2760 (29,4%)	3805 (28%)
Médicaments	6750 (37,9%)	1515 (16,2%)	4132,5 (30,4%)
Consommables	1550 (8,7%)	750 (8%)	1150 (8,5%)
**Total**	**17800**	**9375**	**13587,50**

Taux de change: 1 USD = 910 Fc

Le coût indirect de suivi médical était évalué à 12,3 USD. Il inclut le transport, le manque à gagner pour le patient et le garde malade et les pertes ou vols d'objets constatés à cause du rendez-vous du suivi médical. Le coût total du suivi médical pour les diabétiques pris en charge par les structures du BDOM à Kin-Ouest était donc de 27,2 USD. Le modèle logistique qui explique la non-observance du suivi du diabétique (Tableau 4) n'a retenu que le niveau de vie. Les cas sont à près de six fois plus exposés à un niveau de vie précaire que les témoins (OR= 5,73; CI 95%: 1,58-20,78).

**Tableau 4:** Analyse des déterminants de la non-observance du suivi Clinique du diabète

	OR	95% CI		*P*
Taille ménage	1.09	0.63	1.90	0.749
sexe	1.53	0.70	3.38	0.287
Age	1.20	0.83	1.74	0.330
Connaissance	1.19	0.52	2.71	0.675
Revenu	0.98	0.76	1.26	0.881
Niveau de vie	5.73	1.58	20.78	0.008
Dépenses totales	1.03	0.75	1.43	0.845

La régression logistique multivariée a été utilisée pour étudier les déterminants de l'observance du suivi médical.

## Discussion

Le niveau de vie des ménages est un déterminant majeur de la non-observance. Les non-observants courent six fois plus le risque de provenir d'un ménage pauvre. Une famille nombreuse pourrait prédisposer à la non-observance. Par contre, il n'y a pas d'association entre le niveau de connaissance et la non-observance. L’âge, tout comme le niveau d'instruction, ne jouerait qu'un rôle insignifiant.

Le coût total du suivi médical à la dernière visite était estimé à 27,2 USD, soit 18% du revenu mensuel permanent des ménages (153,17 USD). Le patient sous insuline dépense deux fois plus que celui sous antidiabétiques oraux, lors d'une consultation de suivi.

La non-observance semble être le fait des femmes (63,6%) plus que des hommes (36,4%). Probablement la femme diabétique est plus sollicitée que son collègue homme par de nombreuses autres activités y compris ménagères, l'amenant à ne pas respecter les rendez-vous. Toutefois, étant donné l'appariement par sexe, il n'a pas été possible de dégager l'impact de cette variable dans notre étude. Néanmoins, Babwah et al au Trinidad [18] et Cauch-Dudek et al au Canada [19] ont montré qu'en ce qui concerne le respect de rendez-vous, il n'y a pas de différence entre hommes et femmes.

Le niveau d'instruction ne montre pas une association statistiquement significative avec l'observance du suivi médical. Pourtant on note une différence de niveau d'instruction, telle que les témoins (60,3%) plus que les cas (48,5) ont dépassé le niveau primaire. Toutefois, dans d'autres études, cette association est établie. Les patients avec un bas niveau d'instruction utilisent moins les services de diabète [20]. La taille des ménages n'est pas non plus associée à l'observance. Probablement son effet est contenu dans le niveau de vie. En effet, le revenu faible des ménages est une barrière majeure à l'observance [21]. Le coût total mensuel du suivi médical pour les diabétiques dans les structures du BDOM à Kin-Ouest est estimé à 27,2 USD. Il faut noter que le BDOM a uniformisé les prix des actes pour la prise en charge des diabétiques dans toutes ses structures de soins. Ce coût, 18% du revenu, est sans doute prohibitif pour un patient diabétique qui, en plus, est soumis à un régime alimentaire spécial exigeant du ménage des dépenses supplémentaires. En fait, il constitue un obstacle à l'accès des malades diabétiques aux soins de santé [22]. Il est reconnu que le diabète demeure une affection dont la prise en charge est coûteuse et pour laquelle les patients devraient bénéficier d'une aide particulière [23]. Nous avons estimé le revenu mensuel moyen des ménages des diabétiques à 153,17 USD. Ce chiffre est légèrement inférieur à celui repris par le DSCRP [24] qui le place à 197,34 USD. Cette divergence pourrait suggérer que le diabète appauvrit. Le fait que 72,7% de cas par rapport aux témoins (37,7%) vivent avec moins de 1,25$ par jour en est une autre illustration.

Par ailleurs, les dépenses mensuelles moyennes des ménages sont placées à 306,6 USD, chiffre bien supérieur au revenu mensuel moyen (153,17 USD). Cette dissemblance, peut encore être imputable aux difficultés d'estimation des coûts réels dues au fait d'une économie informelle. Néanmoins, les dépenses mensuelles moyennes par ménage constitueraient un meilleur indicateur du revenu permanent [16]. Voilà pourquoi cette variable a été introduite dans le modèle logistique comme variable dichotomique pour refléter le niveau de vie. Une étude effectuée aux Etats-Unis sur la mortalité chez les diabétiques a montré qu'elle est plus élevée chez ceux vivant en dessous du seuil de pauvreté [21]. Un faible niveau socioéconomique est effectivement reconnu comme un facteur majeur de non-observance du suivi médical. Kapongo, par exemple, a montré que 38,6% des diabétiques ne sont pas réguliers aux rendez-vous de suivi médical suite à de contraintes financières [25]. Les mécanismes par lesquels le faible niveau de vie influe sur l'observance sont multiples. La quasi inexistence de système de partage de coût de santé en RD-Congo fait peser tous le poids de soins sur les patients et leurs familles. Sur 13$ qu'un individu dépense par an pour les soins, 6$ sont payés par la famille, et 2$ seulement par le gouvernement [26]. Les patients sont alors souvent obligés d'opérer le choix de recourir ou pas aux soins de santé en fonction de leurs moyens. Ainsi, les malades démunis auront tendance à attendre que leur condition s'empire avant de se faire soigner [27]. Ou alors ils réduiront le coût de traitement en simplifiant le nombre de prises de médicaments ou de consultations [28]. Il faut redouter que le patient sous insuline, en principe en situation plus précaire, qui dépense deux fois plus que celui sous antidiabétiques oraux puisse réfléchir deux fois avant de se rendre à la consultation de suivi. Le faible revenu des ménages est responsable de 77,3% de cas de non-observance. En d'autres termes, si on améliorait le revenu des ménages des diabétiques, on pourrait prévenir 77,3% de non-observance. Des études ont effectivement montré que l'accompagnement financier des malades améliore l'observance des rendez-vous [29], même si le soutien des pairs [30] et le rappel par le personnel soignant [31] jouent également un rôle important.

Concernant le niveau de connaissance sur le diabète, l’étude montre que 66,2% des cas présentent un faible niveau de connaissance contre 43% des témoins. Kapongo lui a fait le constat de faible niveau de connaissance chez les diabétiques, en général. Il rapporte que 84,7% d'entre eux ont une connaissance médiocre sur le diabète [25]. Comme lui, nous pensons que cette faiblesse serait expliquée partiellement par le faible niveau d'instruction. Il faut noter que le patient est instruit de revenir dans tous les cas ou il l'estime nécessaire notamment en cas de survenue des complications.

Notre étude comporte tout de même quelques limites. Le fait d'avoir utilisé une dichotomisation du revenu, bien que suggérée par le PNUD, fait courir le risque d'une mauvaise classification des revenus qui se situent au centre du continuum. Des ménages, bien qu'ayant un revenu proche, pourraient artificiellement se retrouver dans des classes différentes. En plus, étant donné le fort développement du secteur informel, le revenu des ménages peut avoir été mésestimé. La nature rétrospective de notre étude nous a fait courir le risque de biais de mémoire que nous avons essayé de réduire en prenant des informations sur la dernière visite. Pour évaluer le revenu et les dépenses nous avons considéré la situation au cours du dernier mois précédant l'enquête. Nous aurions pu aussi porter l'appariement sur l’âge pour rapprocher davantage les cas des témoins, ce qui aurait pu nous amener à élargir l'espace de notre étude. Ceci nous a fait courir le risque de biais de sélection étant donné que l’âge pourrait avoir une influence sur l'observance. Néanmoins, le choix des témoins dans la même formation médicale que les cas a sans doute minimisé le biais de sélection.

Un point fort à noter c'est que l'observance n'a pas été définie sur base des déclarations des malades mais plutôt sur vérification de son carnet. Notre approche, considérant une longue période de temps (huit rendez-vous sur 12), a certainement permis de diminuer les cas d'une non-observance accidentelle. Nos résultats méritent d’être approfondis, notamment en élargissant la liste des variables indépendantes. Nous pensons également, qu'ils ne peuvent pas etre applicables dans tous les contextes. C'est, par exemple, le cas du niveau de connaissance du diabète, qui pourrait montrer une association significative dans un autre contexte. Par ailleurs, il y a nécessité de valider la mesure des variables de connaissance sur le diabète.

## Conclusion

L’étude confirme le rôle du bas niveau de vie mais pas celui de l'ignorance comme déterminant de la non-observance des visites de suivi du malade diabétique. Le coût de la prise en charge reste prohibitif pour de nombreux diabétiques. Pour améliorer la régularité des visites, on pourrait considérer l'accompagnement des malades par des activités génératrices de revenu, ou alors, en même temps, promouvoir les mutuelles de santé.
